# Mucormycosis beyond the classic host: a case series and narrative literature review from a tertiary Greek hospital

**DOI:** 10.3389/ffunb.2026.1987890

**Published:** 2026-10-23

**Authors:** Olympia Akritidou, Athanasia – Marina Peristeri, Timoleon-Achilleas Vyzantiadis, Christina-Chrysanthi Theocharidou, Athanassios Kyrgidis, Maria Sileli, Athina Lavrentieva, Anna Nikopoulou

**Affiliations:** 1 Department of Internal Medicine, “G. Papanikolaou” General Hospital of Thessaloniki, Thessaloniki, Greece; 2 Department of Microbiology, Medical School, Aristotle University of Thessaloniki, Thessaloniki, Greece; 3 1^st^ Intensive Care Unit, “G. Papanikolaou” General Hospital of Thessaloniki, Thessaloniki, Greece; 4 Department of Oral & Maxillofacial Surgery, Aristotle University of Thessaloniki, Thessaloniki, Greece; 5 2^nd^ Intensive Care Unit, “G. Papanikolaou” General Hospital of Thessaloniki, Thessaloniki, Greece

**Keywords:** case series, cutaneous mucormycosis, isavuconazole, liposomal amphotericin B, mucorales, mucormycosis, rhino-orbito-cerebral mucormycosis

## Abstract

**Background:**

Mucormycosis is an aggressive, angioinvasive mold infection that progresses rapidly and carries high mortality. Disease in non-classical hosts is readily mistaken for bacterial superinfection or the expected course of the underlying condition and the resulting delay in tissue biopsy and antifungal therapy may adversely affect outcome.

**Methods:**

We report a retrospective case series at a tertiary referral center in northern Greece over a seven-year period.

**Results:**

Nine patients (median age: 49 years, of whom 7 were male and 2 were female) with histopathologically confirmed mucormycosis were reviewed. Two previously healthy adolescents developed post-traumatic cutaneous disease and survived. Three patients reached craniofacial disease through the classic immunosuppressed route, one through poorly controlled type 1 diabetes, one through poorly controlled type 2 diabetes, and one through allogeneic transplantation for acute lymphoblastic leukemia (ALL). The remaining four developed infection on a background of barrier disruption or critical illness: two with major thermal burns (39% and 54% total body surface area), one with a cannulation-site eschar during high-dose corticosteroid therapy for dermatomyositis, and one intubated patient with prediabetes who developed forearm necrosis with local thrombosis. *Rhizopus arrhizus* predominated in the craniofacial and healthcare-associated cases, whereas the trauma and burn cases yielded a wider range of species. Liposomal amphotericin B (L-AmB) was the first-line agent in eight patients and one case with limited disease received isavuconazole monotherapy. Eight patients underwent surgery, ranging from debridement to orbital exenteration, maxillectomy, and in one case forequarter amputation. Eight of nine patients (89%) required admission to the Intensive Care Unit (ICU); among them, the median ICU stay was 37 days (IQR 7-57; range 2-123). Overall mortality was 67% (6/9).

**Conclusions:**

This series includes established metabolic and transplant-associated mucormycosis as well as trauma, burn, and healthcare-associated presentations. Only two patients had no documented systemic predisposition other than traumatic tissue disruption. While this small, heterogeneous patient sample cannot determine incidence, prognostic factors, or treatment effects, it may illustrate that this disease has the potential to affect patients beyond the classic host. Rapidly progressive necrosis or thrombosis should nevertheless prompt consideration of mucormycosis and tissue sampling for histopathology and culture.

## 1 Introduction and clinical significance

Mucormycosis is a rapidly progressive angioinvasive infection caused by fungi of the order *Mucorales*, most often *Rhizopus*, *Mucor*, and *Lichtheimia* species (Jeong et al., 2019b; Liang et al., 2024; Kontoyiannis and Walsh, 2026). Its reported incidence appears to be rising in several regions, and older modelled estimates suggest a prevalence in India up to roughly eighty times that of high-income countries (Prakash and Chakrabarti, 2019; Skiada et al., 2024). Currently, we encounter this disease across every level of hospital care. Diabetes Melitus (DM) remains the dominant predisposing condition globally, accounting for as much as 73% of cases in some cohorts, particularly where glycemic control is poor or ketoacidosis is present (Jeong et al., 2019b; Prakash and Chakrabarti, 2019; Patel et al., 2020). It has also been closely associated with hematologic malignancies, solid organ transplants, chronic corticosteroid usage and severe immunosuppression (Jeong et al., 2019b; Skiada et al., 2025). ICU admission has itself emerged as an independent risk factor, likely through the combined effect of invasive procedures, immunosuppressive therapy, and loss of tissue integrity (Liang et al., 2024). Finally, the COVID-19 pandemic widened this landscape further, as widespread corticosteroid use and hyperglycemia drove a significant surge in cases (Pal et al., 2021; Sen et al., 2021).

Rhino-orbito-cerebral disease is most strongly tied to diabetes, while pulmonary disease predominates in hematology and transplant populations, and cutaneous disease typically follows disruption of the skin barrier by trauma (Jeong et al., 2019b; Skiada et al., 2022; Skiada et al., 2025). Despite growing awareness, clinical reasoning still tends to privilege the classic host. Early signs in patients who do not fit into that category can be very similar to those expected in a case of a bacterial superinfection, an ischemic wound progression, or even the natural course of the disease itself. This similarity in presentation fosters a form of diagnostic anchoring, which could lead to clinician deferral of obtaining a tissue sample for histopathology, thus delaying initiation of antifungal therapy. Delays in diagnosis and source control have been associated with worse outcomes (Chamilos et al., 2008; Cornely et al., 2019; Jeong et al., 2019a).

We describe a seven-year hospital-based experience that includes craniofacial disease in patients with established systemic risk factors and cutaneous disease arising after trauma, burns, or during hospital care. The aim is to report the clinical settings, management, and in-hospital outcomes of these nine cases and to showcase that the disease can arise outside the classical host profile, including -in two of our patients- in the absence of any documented systemic predisposing factor.

## 2 Materials and methods

### 2.1 Study design and setting

This is a retrospective hospital-based series of nine patients treated for mucormycosis at G. Papanikolaou General Hospital of Thessaloniki, a tertiary center serving northern Greece from 1 January 2019 through 31 December 2025. The patients were encountered during routine care and were managed under Infectious Diseases consultation.

### 2.2 Case definition and diagnostic criteria

Invasive mucormycosis was defined by compatible broad, ribbon-like, pauci-septate hyphae invading viable tissue or vessels on tissue biopsy, together with clinical disease at the sampled site (Donnelly et al., 2019). All nine patients met this definition. The reported genus and species assignments were obtained from cultures of relevant clinical specimens. Radiological assessment supported evaluation of disease extent in cases 3-5, 8, and 9. Bronchoalveolar lavage (BAL) culture provided additional microbiological evidence in case 6.

### 2.3 Data collection

The review contained data for all of the patient demographics such as age, sex, comorbidities, admission glycated hemoglobin (HbA1c), corticosteroid exposure, presentation, anatomic site, biopsy findings, culture result, surgery, antifungal treatment, complications, ICU length of stay, and in-hospital outcome. Categorical variables are reported as counts and percentages. Continuous variables are summarized by median, interquartile range (IQR), and range. ICU length of stay was calculated among the eight patients admitted to the ICU. ICU length of stay reflects cumulative ICU days across admissions.

### 2.4 Objectives

The primary objective was to describe the clinical spectrum, management, and in-hospital outcomes of patients with mucormycosis during the study period. A secondary objective was to describe presentations associated with trauma, burns, and healthcare-associated skin disruption. No comparative or causal analysis was planned.

### 2.5 Ethical considerations

This study was conducted in accordance with the Declaration of Helsinki and was approved by the Scientific Council of G. Papanikolaou General Hospital of Thessaloniki (decision of the 20th Regular Session, item 24). Data were extracted retrospectively from existing medical records and handled in pseudonymised form. Written informed consent for the publication of clinical data and potentially identifiable images was obtained from the patients or their legal representatives.

## 3 Results and cases review

### 3.1 Series OVERVIEW

The series included nine patients, seven male and two female, aged 16–55 years. The median age was 49 years. Cases 1–3 had craniofacial disease: two patients had poorly controlled DM, and one had undergone allogeneic hematopoietic stem-cell transplantation (allo-HSCT) for ALL. Cases 4 and 5 were adolescents without a documented systemic immunocompromising condition who developed post-traumatic cutaneous disease. Cases 6 and 7 developed mucormycosis after major burns involving 39% and 54% of total body surface area (TBSA). Cases 8 and 9 developed cutaneous disease during hospital care, one at an intravenous cannulation site while receiving high-dose corticosteroids and one during critical illness. Systemic susceptibility and local tissue injury overlapped in several patients, particularly case 6, who had both poorly controlled diabetes and a major burn. The patients’ baseline characteristics are summarized in **Table 1** below. *Rhizopus arrhizus* predominated in the craniofacial and healthcare-associated cases, whereas the trauma and burn cases yielded a wider range of species. Biopsy sites and culture-confirmed organisms for each patient are summarized in **Table 2**.

**Table 1:** Patient characteristics and clinical setting.

Case	Age/sex	Systemic host factors	Clinical setting and anatomic form	HbA1c	Corticosteroid exposure
1	55/M	T2DM; ESRD	Left mandibular and paranasal craniofacial disease	14.0%	None
2	20/F	T1DM	Right rhino-orbito-cerebral disease	14.5%	None
3	51/F	ALL; allogeneic HSCT	Left rhino-orbital disease	5.0%	Methylprednisolone 40 mg IV q12 h
4	16/M	None documented	Post-traumatic left gastrocnemius wound	5.4%	None
5	17/M	None documented	Post-traumatic left-hand injury with digital amputation	4.6%	None
6	53/M	T2DM; CAD; hypertension	Burn-associated disease; 39% TBSA	13.0%	Hydrocortisone 50 mg IV q6 h
7	54/M	Multiple sclerosis	Burn-associated disease; 54% TBSA	4.3%	Hydrocortisone 50 mg IV q6 h
8	19/M	Dermatomyositis	Cannulation-site cutaneous disease	5.6%	Methylprednisolone 1 g IV daily for 3 days; prednisolone 75 mg daily
9	49/M	Prediabetes; critical illness	Left forearm cutaneous disease with local thrombosis	6.2%	Hydrocortisone 50 mg IV q6 h

ALL, acute lymphoblastic leukemia; CAD, coronary artery disease; ESRD, end-stage renal disease; HSCT, hematopoietic stem-cell transplantation; IV, intravenous; q6 h, every 6 hours; q12 h, every 12 hours; T1DM, type 1 diabetes mellitus; T2DM, type 2 diabetes mellitus; TBSA, total body surface area.

**Table 2:** Case-level diagnostic evidence and culture-confirmed organism.

Case	Biopsy specimen site	Culture-confirmed organism
1	Involved left mandibular and paranasal tissue	*Rhizopus arrhizus*
2	Involved right maxillary and sinonasal tissue	*Rhizopus arrhizus*
3	Involved left orbital and sinonasal tissue	*Rhizopus arrhizus*
4	Involved left gastrocnemius soft tissue	*Rhizopus microsporus*
5	Involved left hand and digital wound tissue	*Lichtheimia ramosa*
6	Involved burn-wound tissue	*Lichtheimia corymbifera;* BAL culture contributed
7	Involved burn-wound tissue	*Mucor circinelloides*
8	Cannulation-site eschar and involved upper-limb tissue	*Rhizopus arrhizus*
9	Necrotic medial left forearm tissue	*Rhizopus arrhizus*

All nine diagnoses were confirmed by tissue biopsy. Genus and species assignments were based on cultures of relevant clinical specimens. BAL, bronchoalveolar lavage.

### 3.2 Craniofacial disease in patients with established systemic risk factors (cases 1–3)

Cases 1–3 had extensive craniofacial disease at diagnosis. One patient had poorly controlled T1DM, one had poorly controlled T2DM, and one had undergone allo-HSCT and remained on corticosteroid treatment.

Case 1. A 55-year-old man with T2DM and end-stage renal disease presented with left mandibular and paranasal oedema (**Figure 1**) and high fever; HbA1c was 14.0%. Imaging defined sinus and craniofacial involvement. Tissue biopsy eventually confirmed invasive mucormycosis, and culture yielded *Rhizopus arrhizus.* Pending results, upon tissue acquisition, L-AmB was started at 10 mg/Kg intravenously every 24 hours. On religious grounds he initially declined debridement surgery, which was ultimately performed four days after the biopsy results confirmed the diagnosis. He underwent left orbital exenteration, radical left maxillectomy, and turbinectomy of the inferior and middle turbinates and remained hospitalized for approximately 20 days before being transferred to a rehabilitation center. Upon subsequent clinical deterioration, he was readmitted emergently in critical condition and transferred to the ICU. His course was complicated by bilateral ischemic infarcts. It was not established whether these resulted from local arterial invasion, thrombosis, or an embolic mechanism. He died after four days in the ICU.

**Figure 1:**
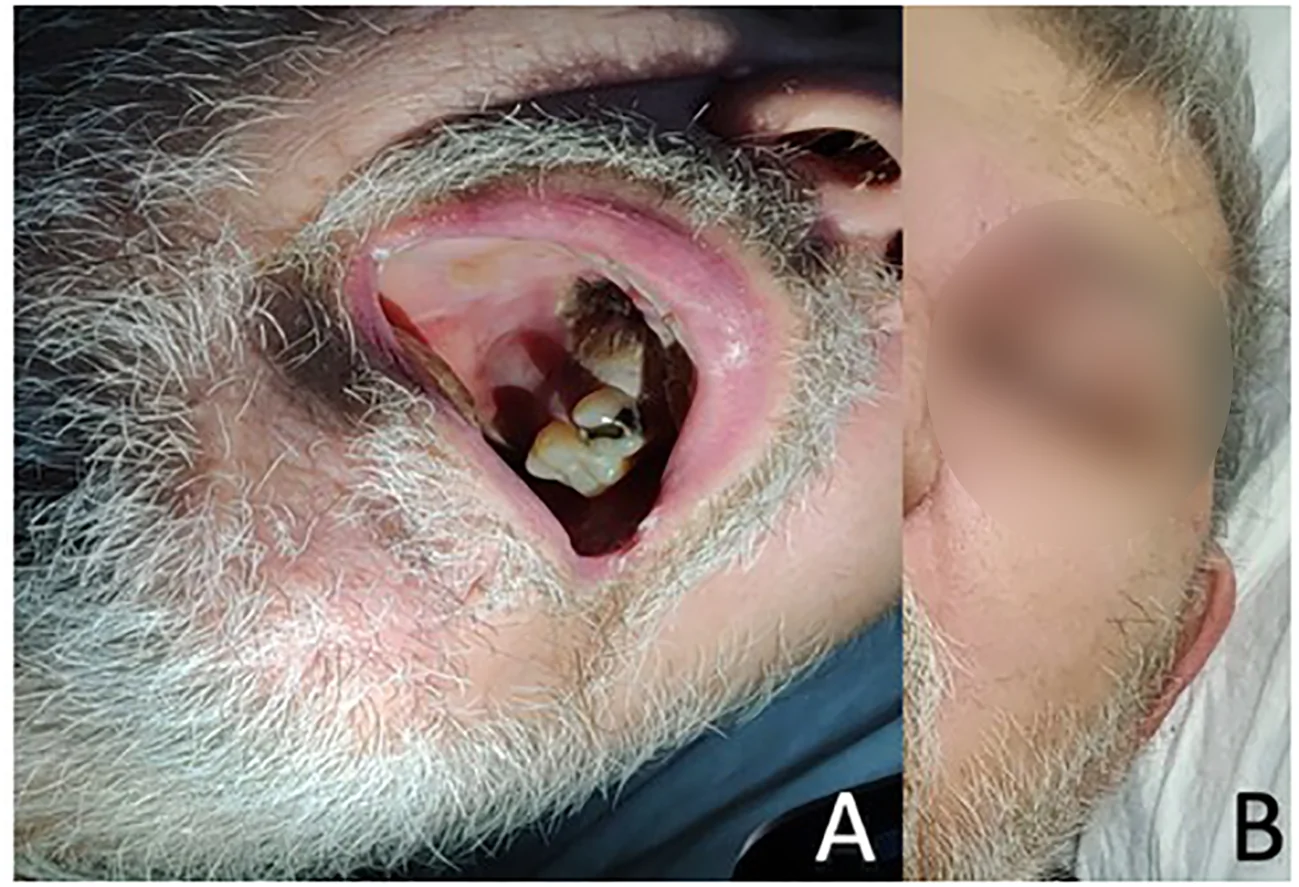
Craniofacial mucormycosis (case 1). **(A)** Necrotic black eschar of the hard palate. **(B)** (Left) periorbital oedema and eyelid swelling at presentation.

Case 2. A 20-year-old woman with T1DM and an HbA1c of 14.5% initially reported toothache. Within days she developed headache, fever, right maxillary swelling, periorbital erythema and a necrotic eschar on the soft palate (**Figure 2**). Given the high clinical suspicion of invasive mucormycosis, L-AmB (10 mg/kg intravenously every 24 hours) was started pending biopsy and culture results. Imaging (**Figure 3**) and tissue biopsy confirmed rhino-orbito-cerebral disease, and culture yielded *Rhizopus arrhizus* five days later, with a moderately elevated amphotericin B MIC of 4 mg/L (EUCAST broth microdilution, reference mycology laboratory). In view of this MIC and the extent of her disease, and in the absence of clinical breakpoints for *Mucorales*, isavuconazole (loading dose of 200mg every 8 hours for six doses and then 200 mg once daily) was added as an individualized decision based on both findings, in line with the strong recommendation for triazoles as salvage therapy (Cornely et al., 2019).On day 6 of antifungal therapy, following the positive culture result, the patient underwent surgery, which consisted of right orbital exenteration, excision of the adjacent right ethmoidal sinus walls, segmental mandibulectomy, and partial sphenoid sinus resection. Nonetheless, rhinocerebral extension resulted in coma requiring intubation, and vascular invasion caused right internal carotid artery thrombosis with basal ganglia infarcts. Her prolonged intensive care course was complicated by ventilator-associated pneumonia and *Clostridioides difficile* infection. Once stable, she was transitioned to oral isavuconazole (200 mg once daily) and, after a 123-day ICU stay (the longest in our series), she was discharged alive. Following discharge, she underwent reconstructive plastic surgery and has since recovered fully, remaining in good health with no lasting sequelae.

**Figure 2:**
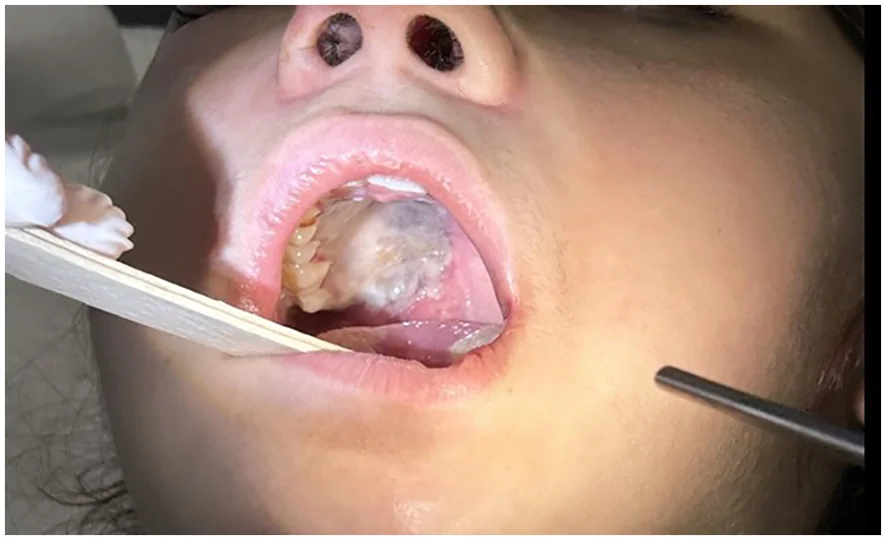
Rhinocerebral mucormycosis in a patient with DM type 1 (case 2).

**Figure 3:**
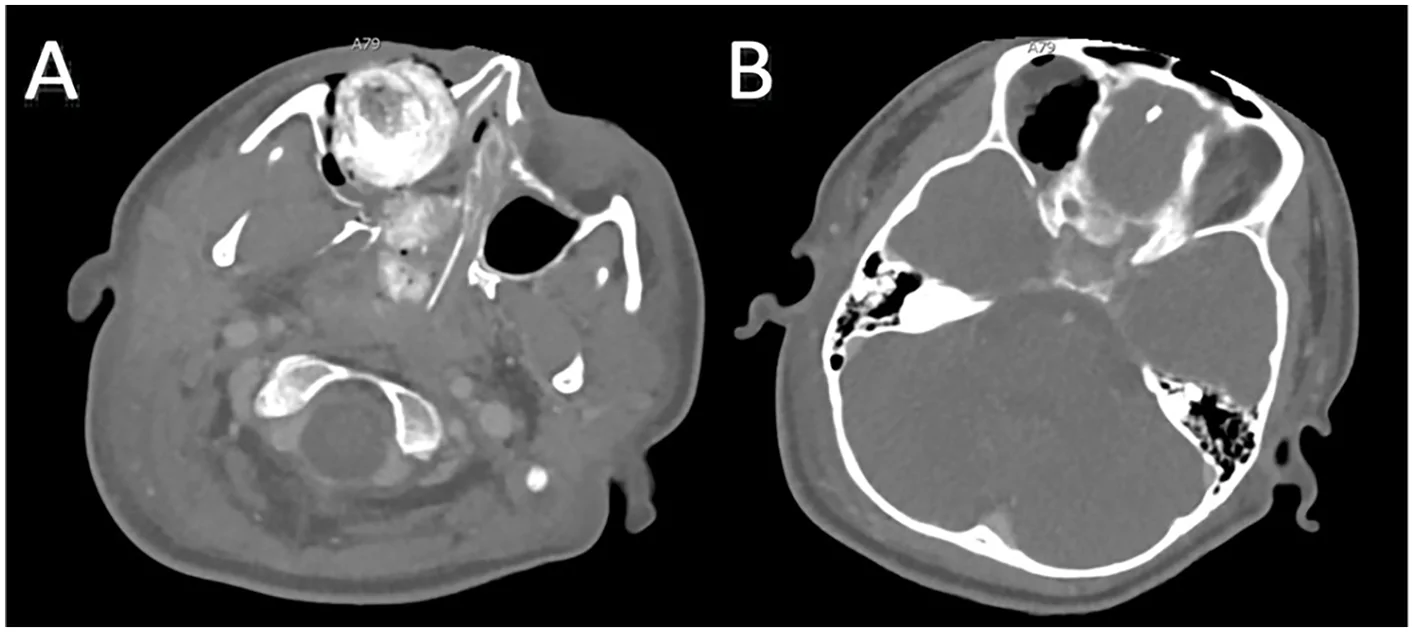
Case 2, pre **(A)** and post **(B)** -operatively, after right orbital exenteration: abnormal enhancing soft tissue abutting the lateral right orbital wall.

Case 3. A 51-year-old woman with ALL had undergone allo-HSCT and was receiving methylprednisolone 40 mg intravenously every 12 hours. She presented with left periorbital oedema and worsening visual impairment, and further imaging defined the extent of her disease. L-AmB 10mg/kg intravenously every 24 hours was initiated on the day of presentation without awaiting mycological confirmation. Because the severity of her immunosuppression left little host capacity to contain an angioinvasive infection, surgical source control was considered urgent and she underwent left orbital exenteration on day 3. Imaging and tissue biopsy subsequently confirmed rhino-orbital mucormycosis, and culture yielded *Rhizopus arrhizus*. Post-operative imaging is shown in **Figure 4**. She was managed postoperatively in the ICU but deteriorated rapidly, and septic shock culminated in a fatal cardiac arrest on the eighth day of her ICU stay.

**Figure 4:**
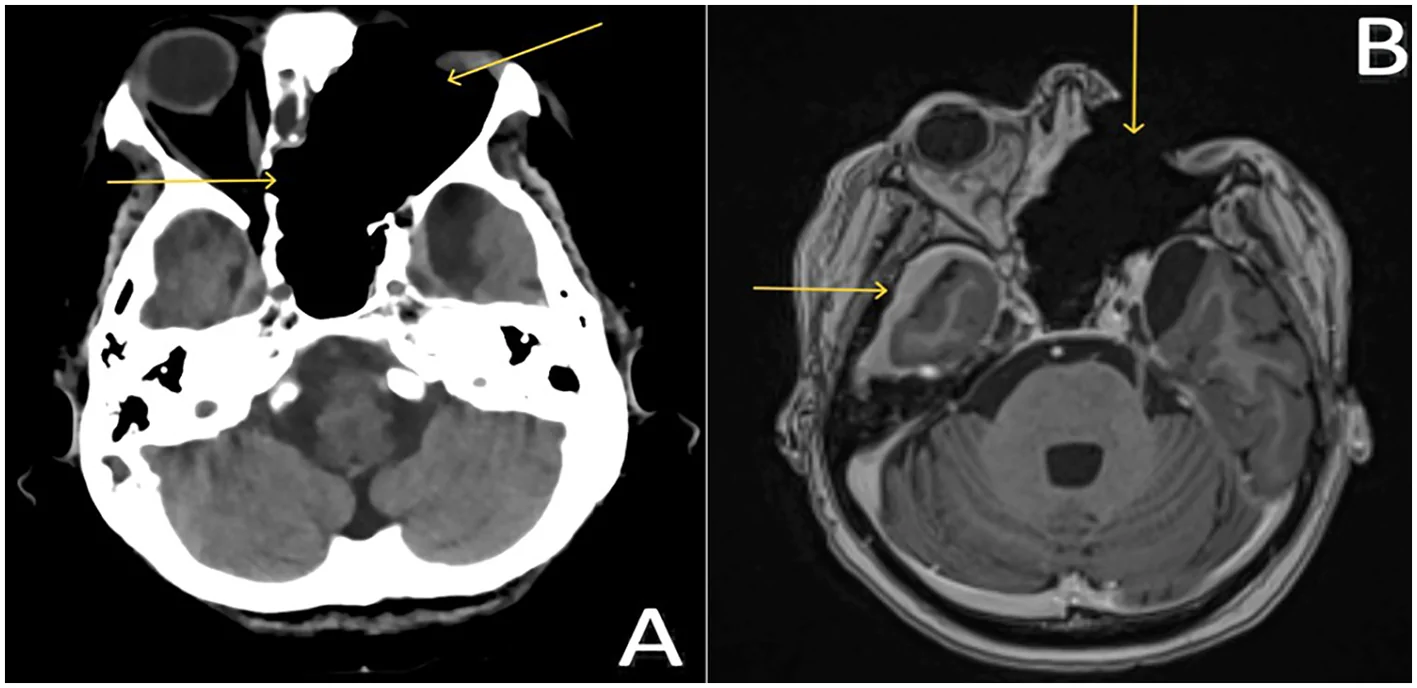
Case 3, post-operatively. Axial **(A)** CT and **(B)** MRI showing the surgical defect after left orbital exenteration and debridement of the affected adjacent bone and soft tissues.

### 3.3 Trauma in previously healthy hosts (cases 4–5)

Cases 4 and 5 were adolescents without a documented immunocompromising condition or any known metabolic risk factor. In both, invasive disease followed traumatic disruption of skin and soft tissue, providing a direct route of inoculation into injured tissue. Both underwent debridement, received antifungal treatment, and survived to discharge without documented lasting effects.

Case 4. A 16-year-old boy presented with a post-traumatic wound over the left gastrocnemius, with localized erythema, tenderness, and fever. He had no recorded comorbidity, and HbA1c was 5.4%. He underwent debridement on day 2 and tissue was sent for histopathology and culture. Because he was not immunosuppressed and the disease appeared localized, antifungal therapy was not started empirically. Biopsy of involved tissue confirmed invasive mucormycosis, and culture yielded *Rhizopus Microsporus* on day 6. Consequently, he received L-AmB 5 mg/kg intravenously every 24 hours. After clinical improvement he was switched to oral isavuconazole receiving a loading dose of 200mg every 8 hours for six doses and then 200 mg once daily for a total of 4 weeks, until clinical resolution. He did not require ICU admission and was discharged alive.

Case 5. A 17-year-old boy sustained a left-hand injury with traumatic digital amputation. He had no recorded comorbidity, and HbA1c was 4.6%. He underwent surgery on day 1 and tissue was sent for histopathology and culture. As in case 4, he was not started on empirical antifungal therapy. Tissue biopsy confirmed invasive mucormycosis, and culture yielded *Lichtheimia ramosa* on day 4. Monotherapy with oral isavuconazole was initiated on the same day (loading dose of 200mg every 8 hours for six doses and then 200 mg once daily) which he received for 4 weeks, until the amputation stump had healed without signs of recurrent necrosis. He was discharged alive after a two-day ICU stay.

Both isolates, *Rhizopus microsporus* and *Lichtheimia ramosa*, are environmental *Mucorales* capable of causing invasive cutaneous infection after traumatic inoculation, including in patients without documented systemic immunosuppression (Kronen et al., 2017; Skiada et al., 2022). In these cases, invasion followed loss of the physical tissue barrier.

### 3.4 Burn-associated disease (cases 6–7)

Cases 6 and 7 developed mucormycosis after major thermal injury. Both had extensive devitalized tissue, developed septic shock, received hydrocortisone, and died after prolonged intensive care. The available records do not establish when fungal invasion began relative to their systemic deterioration.

Case 6. A 53-year-old man with T2DM, coronary artery disease (CAD) and hypertension sustained burns involving 39% TBSA when an unexploded artillery shell detonated accidentally while he was digging for gold. The wounds were heavily contaminated with soil and debris. HbA1c was 13.0%. He received hydrocortisone 50 mg intravenously every 6 hours for septic shock. Burn-wound tissue biopsy showed invasive mucormycosis and the burn-wound culture became positive for *Lichtheimia corymbifera* on day 7, when L-AmB 10mg/kg intravenously every 24 hours was started. BAL culture also yielded *Lichtheimia corymbifera*. The respiratory isolate provided additional microbiological evidence, but the available data could not determine whether it represented pulmonary disease, airway presence, or the route of dissemination. No surgical debridement was undertaken for mucormycosis, given the difficulty of operating on a critically ill patient with such extensive burns, which left the fungal burden without mechanical source control. His clinical course was characterized by severe sepsis, which gave rise to acute kidney injury necessitating haemodialysis and a non-ST-elevation myocardial infarction, before culminating in multiorgan failure and a fatal outcome on the 42nd day of intensive care.

Case 7. A 54-year-old man with a prior history of multiple sclerosis sustained burns involving 54% TBSA. HbA1c was 4.3%, and he received hydrocortisone 50 mg intravenously every 6 hours during septic shock. Burn-wound tissue biopsy showed invasive mucormycosis, and culture yielded *Mucor circinelloides* on day 12 of hospitalization, upon which L-Amb 10mg/kg was initiated intravenously every 24 hours. He subsequently underwent serial debridement with split-thickness skin grafting. The admission was complicated by herpes zoster ophthalmicus and severe Gram-negative sepsis, which progressed to multiorgan failure. He died after 44 days in the ICU.

In both patients, tissue necrosis, fever, hemodynamic instability, and raised inflammatory markers could initially have been attributed to the burn or to bacterial infection. From a clinical standpoint, a burn wound that becomes disproportionately necrotic, develops a thrombotic appearance, or fails to respond as expected to antibacterial treatment warrants tissue sampling for histopathology and fungal culture.

### 3.5 Healthcare-associated cutaneous disease: instrumentation as a portal of entry (cases 8–9)

Cases 8 and 9 developed cutaneous mucormycosis during hospital care. Case 8 was already immunosuppressed by high-dose corticosteroid treatment for dermatomyositis, and the lesion arose at a documented intravenous cannulation site. Case 9, by contrast, had no comparable predisposition. In this case, critical illness and a cutaneous portal that was not specifically documented converged on the same outcome.

Case 8. A 19-year-old man with dermatomyositis receiving pulse methylprednisolone (1 g IV over three days) and maintenance prednisolone (75 mg daily; HbA1c 5.6%) developed a necrotic eschar at an intravenous cannulation site on hospital day 7 (**Figure 5A**). As the lesion progressed, tissue was obtained for histopathology and culture on day 10 and L-AmB (10 mg/kg q24h IV), was started empirically on the same day. On day 14, histopathology confirmed cutaneous *Rhizopus arrhizus* infection. Initial debridement was performed that day (**Figure 5B**). Despite antifungal therapy and debridement, the infection extended proximally, thus necessitating left forequarter amputation on day 20, the most extensive in the series. He was diagnosed with anti-MDA5 dermatomyositis, a phenotype associated with rapid disease progression and high short-term mortality. Due to disease progression, he developed respiratory failure that required mechanical ventilation. He was subsequently admitted to the ICU, where his course was complicated by ventilator-associated pneumonia. He succumbed after a 95-day course in the ICU.

**Figure 5:**
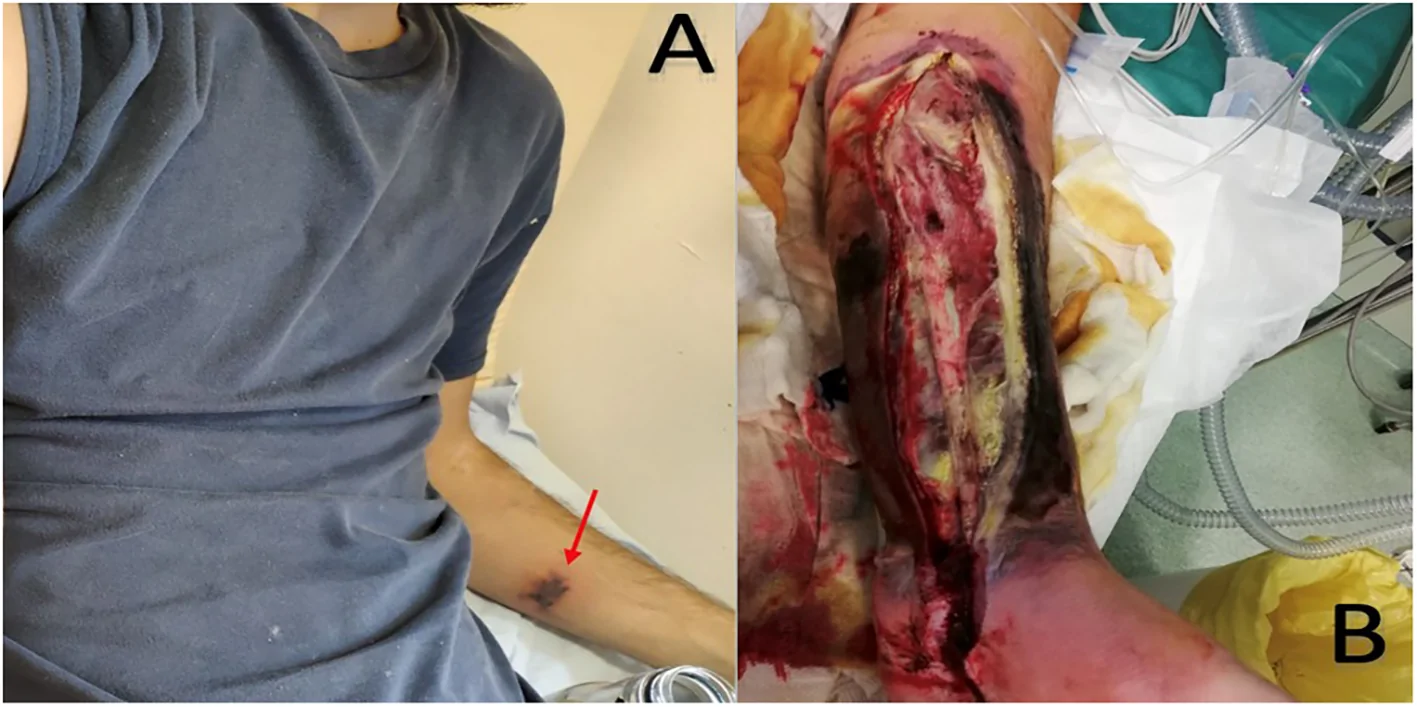
Cutaneous mucormycosis at IV cannulation site (case 8). **(A)** Initial necrotic eschar. **(B)** Post-debridement appearance.

Case 9. A 49-year-old man with prediabetes and depression was admitted and intubated for neuroleptic malignant syndrome. During his ICU stay he developed acute respiratory distress syndrome and Gram-negative sepsis and received hydrocortisone 50 mg intravenously every 6 hours. On ICU day 5, cutaneous necrosis with local thrombosis subsequently developed over the medial left forearm. Tissue was obtained for histopathology and culture on day 6. On day 10, the results confirmed invasive mucormycosis and culture yielded *Rhizopus arrhizus*. He was then initiated on L-AmB (10 mg/kg IV q24h) and underwent debridement on day 12. His course was complicated by empyema and a fatal cardiac arrest after 32 days.

### 3.6 Treatment and in-hospital outcomes

Immediate surgical assessment for debridement was prioritized whenever anatomically and physiologically possible, alongside systemic antifungal therapy. L-AmB was the initial antifungal of choice in eight patients, at 10mg/kg q24h in those with extensive or rapidly progressive disease and at 5mg/kg q24h in one patient with localized cutaneous disease (case 4). Isavuconazole was used as monotherapy in case 5 (localized disease) and as part of combination therapy (case 2) and as oral step-down therapy in case 4.

Surgical treatment ranged from local debridement to orbital exenteration, maxillofacial resection, skin grafting, and forequarter amputation. Eight of nine patients (88.9%) required ICU care. Among these eight, the median ICU stay was 37 days (IQR 7-57; range 2-123). Six patients died in hospital (66.7%). Antifungal treatment, surgical procedures, ICU length of stay, and in-hospital outcome for each patient are summarized in **Table 3**.

**Table 3:** Antifungal treatment, surgery, ICU stay, and in-hospital outcome.

Case	Antifungal treatment	Surgery	ICU stay	In-hospital outcome
*1*	L-AmB 10 mg/kg IV q24 h	Left orbital exenteration; radical left maxillectomy; inferior and middle turbinectomy	4 d	Death
*2*	L-AmB 10 mg/kg IV q24 h + isavuconazole 200 mg once daily (combination)	Right orbital exenteration; right ethmoidal wall excision; segmental mandibulectomy; partial sphenoid sinus resection	123 d	Discharged alive
*3*	L-AmB 10 mg/kg IV q24 h	Left orbital exenteration	8 d	Death
*4*	L-AmB 5 mg/kg IV q24 h; oral isavuconazole 200 mg once daily step-down	Debridement	No ICU	Discharged alive
*5*	Oral isavuconazole 200 mg	Debridement	2 d	Discharged
*6*	L-AmB 10mg/kg IV q24 h	No mucormycosis-directed debridement	42 d	Death
*7*	L-AmB 10 mg/kg IV q24 h	Serial debridement; split-thickness skin grafting	44 d	Death
*8*	L-AmB 10 mg/kg IV q24 h	Debridement; left forequarter amputation	95 d	Death
*9*	L-AmB 10 mg/kg IV q24 h	Debridement	32 d	Death

d, days; ICU, intensive care unit; IV, intravenous; L-AmB, liposomal amphotericin B; q24 h, every 24 hours.

## 4 Discussion and review of the literature

Mucormycosis is still taught, and still instinctively recognized, as a disease of the poorly controlled diabetic and the profoundly immunocompromised. Yet the biology that permits *Mucorales* to invade is more permissive than that teaching implies: these are ubiquitous environmental molds, and angioinvasive disease requires only that a sufficient inoculum reaches devitalized, poorly perfused tissue in a host whose phagocytic defenses are overwhelmed, whether locally or systemically (Ibrahim et al., 2012; Liang et al., 2024; Kontoyiannis and Walsh, 2026). In the discussion that follows we set the series against the contemporary literature, and develop a single argument: that outcome in mucormycosis is governed jointly by the host’s immune competence and by the speed with which infection is suspected, confirmed, and brought under surgical and antifungal control (Cornely et al., 2019; Jeong et al., 2019a; Kontoyiannis and Walsh, 2026). Host immune status is largely fixed at presentation, but the timeliness of recognition is not. In the non-classical host, where suspicion is lowest, it could yet be this modifiable delay that may help separate a salvageable patient from a fatal course.

### 4.1 Epidemiology

The reported mucormycosis burden and host-factor distribution vary substantially by region. An older modelled estimate placed the prevalence in India at approximately 80 times that reported in high-income countries, a difference closely linked to the burden of poorly-controlled diabetes (Prakash and Chakrabarti, 2019). In an extensive systematic review of published cases by Jeong et al, diabetes was the most common underlying condition and was strongly associated with rhino-orbito-cerebral disease, while hematological malignancy and transplantation were more often linked to pulmonary or disseminated infection (Jeong et al., 2019b).

The ECMM/ISHAM Zygomyco.net registry further illustrates this regional variation. Across 16 countries, rhino-orbito-cerebral, pulmonary, and cutaneous disease accounted for 36.6%, 33.2%, and 17.5% of reported cases, respectively, but the distribution of host factors differed markedly between national cohorts (Skiada et al., 2025). European analyses also suggest that reported incidence is increasing, although changes in awareness, diagnostic capacity, surveillance, and the population at risk make it difficult to distinguish a true rise in disease from improved case ascertainment (Prakash and Chakrabarti, 2019). In a multicenter, nationwide prospective survey conducted in Greece from 2005 to 2022, the annual incidence was estimated to be stable after 2009 at 0.54 cases per million population. Greek surveillance has likewise shown heterogeneous anatomic forms and risk profiles (Drogari-Apiranthitou et al., 2023).

There is a possible circularity, which we offer as a hypothesis. Clinicians anticipate (or at least have come to expect) mucormycosis as an infection found primarily in diabetic patients and those with neutropenia. Therefore, they will look for it, sample it, and document it in diabetic and neutropenic individuals and to a lesser degree or less frequently in burn patients, injured patients, and critically ill patients, whose necrotic tissue invites simpler explanations. Therefore, while the clinical epidemiology of the diabetic host may provide a framework for anticipating the likelihood of encountering mucormycosis, it has also provided a mechanism whereby the hosts in whom the illness is most difficult to recognize will be under-represented on the clinical epidemiologic “map” based upon how we identify, sample and document infections. What this case series from our institution does however is compile into a singular reference, the presentations of mucormycosis that are most likely to escape identification.

### 4.2 Pathophysiology

The clinical heterogeneity of mucormycosis reflects several routes to a common invasive process. *Mucorales* spores are ubiquitous in the environment and are normally contained by intact epithelial barriers and phagocytic host defenses. Disease becomes more likely when these defenses are impaired systemically, as in neutropenia, corticosteroid exposure, or severe metabolic disturbance, or locally, when trauma or burns introduce spores directly into poorly perfused and devitalized tissue (Ibrahim et al., 2012; Liang et al., 2024; Kontoyiannis and Walsh, 2026).

Once germination occurs, the hallmark is angioinvasion: hyphae penetrate and proliferate within blood-vessel walls, resulting in thrombosis, infarction, and the dry, necrotic, poorly perfused tissue that is the clinical signature of the disease. This vascular destruction renders the infected bed avascular and therefore largely inaccessible to systemically administered antifungal drugs, which provides a rationale for surgical debridement in addition to systemic antifungal therapy (Ibrahim et al., 2012; Kontoyiannis and Walsh, 2026).

At the molecular level, endothelial invasion is mediated in large part by the fungal spore-coat protein family CotH, which is uniquely expanded in *Mucorales* and binds the host receptor GRP78 (glucose-regulated protein 78) on endothelial and nasal epithelial cells, triggering endocytosis, host-cell damage, and hematogenous dissemination (Alqarihi et al., 2020; Alanazi et al., 2026). Interestingly, the conditions of diabetic ketoacidosis (i.e. hyperglycemia, an acidic pH, elevated free iron, and circulating ketone bodies) upregulate both GRP78 and CotH3, amplifying invasion (Alqarihi et al., 2020). Iron availability is itself a decisive permissive factor. In ketoacidosis, acidemia displaces iron from transferrin and increases the pool of free iron, which *Mucorales* acquire efficiently through a reductive uptake pathway centered on the high-affinity iron permease Ftr1, a virulence determinant in animal models (Ibrahim et al., 2012). Their endogenous siderophore, rhizoferrin, obtains iron from serum inefficiently. Instead, *Mucorales* exploit deferoxamine as a xenosiderophore, which explains why iron overload, including transfusion-related overload, and deferoxamine therapy are classical risk factors (Ibrahim et al., 2012; Alqarihi et al., 2020; Liang et al., 2024). Meanwhile, trauma, burn, and device-associated cases illustrate a complementary mechanism, in which a physical breach delivers a large environmental inoculum directly into devitalized tissue, and corticosteroid exposure or critical-illness immunoparalysis removes the phagocytic containment that would otherwise clear it. In other words, the diabetic host and the burned or traumatized host arrive at angioinvasive mucormycosis by different routes but converge on the same destructive endpoint (Ibrahim et al., 2012; Liang et al., 2024).

### 4.3 The classical hosts in context (cases 1–3)

Rhino-orbito-cerebral disease is the form most strongly tied to diabetes and the dominant presentation worldwide, and in the poorly-controlled diabetic it behaves with the rapid angioinvasive, thrombotic progression that defines the disease’s reputation (Jeong et al., 2019b; Liang et al., 2024). True to that, all three craniofacial cases in our series (Cases 1–3) presented with rhino-orbital or rhino-orbito-cerebral disease, as this literature would predict, and the sole survivor among them (Case 2) was young, had a correctable metabolic derangement, and underwent radical surgical source control with antifungal therapy; population studies report associations between such factors and survival but cannot establish causation in an individual patient (Sigera and Denning, 2024; Zia et al., 2025).The hematologic host follows a different natural history: registry and cohort data link malignancy and transplantation with pulmonary and disseminated rather than craniofacial disease, and with a higher mortality reflecting neutropenia, mucosal-barrier injury, and continuing immunosuppression (Cui et al., 2025; Skiada et al., 2025). As such, when rhino-orbito-cerebral disease does arise in this group, it tends to be more refractory than its diabetic counterpart. The place of surgery diverges accordingly: in hematologic patients with rhino-orbito-cerebral disease, the absence of surgical resection is an independent predictor of early death, with one cohort reporting 25% mortality after combined surgical and antifungal therapy against 58.3% with medical therapy alone (Chen et al., 2023). Yet the same literature carries a sobering qualification: in the profoundly immunosuppressed host, even prompt and aggressive source control may not suffice, because the deficit that permits invasion, the failure of phagocytic clearance, is the one variable surgery cannot correct.

### 4.4 Trauma in the immunocompetent host (cases 4–5)

The two trauma-associated patients were the only individuals in the series with no identifiable immune or metabolic defect, and both survived. Their disease arose purely from inoculation of environmental *Mucorales* through traumatic disruption of the skin and soft tissue, a mechanism well characterized in the trauma literature. Post-traumatic invasive fungal infection is recognized to occur in fully immunocompetent hosts when a sufficient inoculum is introduced into devitalized tissue, and it is caused by a more heterogeneous group of *Mucorales* than the *Rhizopus*-dominated craniofacial disease of the diabetic host (Kronen et al., 2017; Skiada et al., 2022). This is also mirrored in our series: the trauma cases yielded *Rhizopus microsporus* and *Lichtheimia (Absidia) ramosa*, environmental molds that rarely cause invasive disease in the absence of barrier disruption (Skiada et al., 2022). Population-level data reinforce the clinical relevance of this route (Clark et al., 2023). The trauma literature also quantifies the diagnostic delay that recurs throughout this discussion: in military series, intervals of several days from injury to first positive culture and one to two weeks to initiation of antifungal therapy have been documented (Warkentien et al., 2012; Weintrob et al., 2015). Both of our trauma patients survived to discharge. Preserved immunity and surgical debridement may have contributed, although with only two patients this remains a possible explanation rather than an inferred cause. Their course is nonetheless consistent with the observation that localized cutaneous disease in an immunocompetent host carries the most favorable prognosis in the entire spectrum of mucormycosis (Skiada et al., 2022; Sigera and Denning, 2024).

### 4.5 Burn-associated mucormycosis (cases 6–7)

Both burn-associated patients died. Burn wounds create a particularly difficult setting for recognition because devitalized tissue, fever, hemodynamic instability, and elevated inflammatory markers are expected, and bacterial superinfection is common. *Mucorales* may therefore be detected only after invasion is advanced, as is documented in most cases (Littlehales et al., 2022; Dang et al., 2023).

A systematic review of post-burn mucormycosis by Dang et al. identifies burn extent and multifocal involvement as the dominant predictors of death: a TBSA above 50% carries the greatest prognostic weight, and disseminated disease reaches 80% mortality against 36% for localized infection (Dang et al., 2023). In the same review, diabetes was not significantly associated with mortality once a major burn was present; a non-significant association is not proof of no effect, though case 6 (a poorly-controlled diabetic whose infection nonetheless entered through the burn) is consistent with a predominant role for the wound. The harder problem is recognition. Burned tissue is expected to slough, critical illness to produce fever and instability, and bacterial superinfection to drive inflammatory markers, so *Mucorales* hide within an entirely plausible clinical picture and are typically identified only once invasion is advanced (Littlehales et al., 2022).

A bi-center French study of systematic weekly screening for circulating *Mucorales* DNA in severely burned patients reduced the time to diagnosis by eight days and to treatment initiation by 3.5 days compared with conventional mycological methods, and was associated with a reduction in 100-day mortality from 63.6% to 30.8% (overall P = 0.071; significant only in the subgroup with burns of at least 30% TBSA, P = 0.047) (Faure et al., 2025). Because screening was combined with earlier treatment and surgery, the survival difference cannot be attributed to earlier diagnosis alone. Case 6 is also instructive for a second reason: no surgical debridement was undertaken, owing to the difficulty of operating on a critically ill patient with extensive burns. In a disease in which combined surgical and antifungal therapy is repeatedly associated with markedly better survival than antifungal therapy alone, the absence of source control may have limited the effectiveness of antifungal therapy against a mold embedded in avascular, devitalized tissue (Jeong et al., 2019a; Dang et al., 2023; Sigera and Denning, 2024).

### 4.6 Healthcare-associated disease (cases 8–9)

The final two cases mark the furthest extension of the non-classical host, in which infection is acquired not despite medical care but through it. Both belong to the recognized category of healthcare-associated mucormycosis, in which the portal of entry is iatrogenic and the predisposition is the treatment or its setting, corticosteroids and critical illness, rather than any underlying hematologic or metabolic disease; the reported spectrum ranges from device- and procedure-related cutaneous infection to peritoneal mucormycosis complicating peritoneal dialysis (Rammaert et al., 2012). The cutaneous route is the one most directly tied to instrumentation, with nosocomial and post-traumatic cutaneous mucormycosis well characterized (Simbli et al., 2008; Bonifaz et al., 2012; Skiada et al., 2022) and reported even after repeated minor trauma at insulin-injection sites (Chambers et al., 2011). Our two cases sit squarely within this spectrum: in Case 8, an intravenous cannulation site in a corticosteroid-immunosuppressed host, and in Case 9, cutaneous disease during intensive-care support without a specifically documented portal of entry; both yielded cutaneous *Rhizopus arrhizus*. This is the category clinicians are least primed to suspect, and although the evidence base remains thin it is growing, with series describing mucormycosis in critically ill patients, most of whom were nonetheless immunosuppressed, and its high mortality once established in the ICU (Rothe et al., 2021; Yang et al., 2025).

### 4.7 Diagnosis

Every pathway traced so far converges on a single clinical act, which is obtaining tissue, and doing so early. Histopathology remains the diagnostic gold standard and confirmed all nine of our cases, showing broad, ribbon-like, pauciseptate hyphae with wide-angle branching and angioinvasion; compatible histology supports invasive mucormycosis, but culture or molecular confirmation is required where feasible, particularly when morphology is equivocal (Skiada et al., 2020). Yet histopathology depends on the very act this series argues is too often deferred, i.e. obtaining tissue, and culture, the traditional means of species identification, is insensitive, growing the organism in only around half of proven cases (Zacharias et al., 2024; Skiada et al., 2020).

The field has moved substantially in this interval. Combined histopathology and internal-transcribed-spacer (ITS) sequencing improves species-level identification from formalin-fixed tissue (Zacharias et al., 2024). More transformative has been the development of *Mucorales*-specific quantitative PCR. The prospective MODIMUCOR trial, evaluating serum *Mucorales* qPCR in 232 patients, reported a sensitivity of 85.2% and specificity of 89.8%, and, most importantly for the argument of this paper, found the first positive serum PCR a median of four days before the first positive mycological or histological specimen and one day before the first radiological sign (Millon et al., 2022). *Mucorales* PCR on BAL fluid extends molecular detection to pulmonary disease, with high specificity in a small study. Because the number of confirmed cases was very limited, the sensitivity estimate is imprecise and negative results should be interpreted with caution (Prattes et al., 2026). Newer non-invasive approaches, including plasma Mucorales cell-free DNA PCR (Mah et al., 2025; Mah et al., 2026) and metagenomic next-generation sequencing of peripheral blood (Wang et al., 2024) and antigen-based serology using *Rhizopus arrhizus* antigens (Choudhary et al., 2024) are expanding the diagnostic repertoire further. The burn-screening study discussed above found that deploying molecular detection pre-emptively was associated with earlier treatment and lower mortality, although its design cannot confidently establish that screening alone caused the survival difference (Faure et al., 2025). Whether earlier molecular testing would have altered outcomes in our patients cannot be determined, as it was not performed.

The practical synthesis is that, in the non-classical host, the clinical signal that should trigger this diagnostic cascade is consistent: necrosis that is disproportionate, thrombogenic, rapidly progressive, or unresponsive to antibacterial therapy is not adequately explained by the background diagnosis and demands tissue biopsy, with molecular testing as an increasingly available adjunct, regardless of whether a classical predisposing condition is present (Cornely et al., 2019; Skiada et al., 2020; Skiada et al., 2022).

### 4.8 Imaging

Imaging cannot establish the diagnosis, as no finding is specific for *Mucorales*, but it defines the anatomical extent of disease, directs the reach of surgery, and increasingly contributes to early suspicion (Skiada et al., 2020; Lamoth et al., 2024). On computed tomography, the reverse halo sign is among the more suggestive early features of pulmonary disease, although it occurs in other angioinvasive molds and is neither sensitive nor specific (Skiada et al., 2020; Lamoth et al., 2024). In rhino-orbito-cerebral disease, magnetic resonance imaging better delineates the features that determine both prognosis and the extent of resection, perineural and perivascular spread, cavernous-sinus involvement, and cerebral infarction (Sehgal et al., 2022). Functional imaging is an emerging adjunct: FDG-PET/CT can localize occult or multifocal foci and, by quantifying metabolic activity, has been explored as an adjunct for detecting multifocal disease and monitoring metabolic activity, although its incremental clinical value remains uncertain (Phulia et al., 2025).

### 4.9 Treatment: source control, antifungal selection, and adjunctive approaches

The effective management of mucormycosis rests on three pillars: aggressive surgical debridement, prompt antifungal therapy and reversal of the underlying predisposition. The pre-eminence of surgery is well established: pooled analysis associates combined surgical and antifungal therapy with an approximately four-fold reduction in the odds of death relative to antifungal therapy alone (Jeong et al., 2019a) a finding reproduced across the haematologic and burn literatures (Chen et al., 2023; Dang et al., 2023). Current ECMM/MSGERC guidance strongly recommends high-dose L-AmB (5–10 mg/kg daily) as first-line therapy, with isavuconazole and posaconazole recommended as alternatives of moderate strength, alongside early surgery and control of the host condition (Cornely et al., 2019; Kontoyiannis and Walsh, 2026). Isavuconazole has also become established as oral step-down therapy: a systematic review of 135 adults reported all-cause mortality of 27.4% at 42 days, with favorable outcomes when induction with L-AmB was followed by oral isavuconazole maintenance, although adverse-event data were incompletely reported (Gunathilaka et al., 2025). We used isavuconazole as step-down therapy in case 4 and as monotherapy in case 5, although whether isavuconazole monotherapy can be generalized to cutaneous-only disease in immunocompetent hosts remains unproven and warrants prospective study. Evidence remains insufficient to support routine combination therapy or adjunctive immunotherapy. Intrinsic azole resistance in *Rhizopus arrhizus* has a defined molecular basis and constrains oral options, reinforcing the value of species-level identification and susceptibility testing (Lackner et al., 2026). In case 2, isavuconazole was added as an individualized decision, in view of a moderately elevated amphotericin B MIC and extensive disease. Nevertheless, despite its intuitive appeal, combination antifungal therapy has not been shown to improve survival over monotherapy (Jeong et al., 2019a; Meena et al., 2023). What has drawn more interest is the timing of step-down, with Coste et al. studying how long L-AmB should run before changing to a triazole (Coste et al., 2026). Adjunctive immunotherapy (interferon-γ and immune-checkpoint inhibition with anti-PD-1 agents to reverse the immunoparalysis of invasive mold infection) is supported at present only by small, uncontrolled experiences and must be regarded as experimental (Serris et al., 2026). Recombinant human granulocyte-macrophage colony-stimulating factor (rhu GM-CSF; e.g., sargramostim), which augments the oxidative burst, phagocytosis, and hyphal-damaging activity of neutrophils and monocytes, including enhancement of neutrophil-mediated damage to *Mucorales in vitro*, has been explored on the same experimental basis (Chen et al., 2022). In a single-center series combined with a systematic review, *Mucorales* accounted for 13 of 50 collated cases of invasive fungal disease treated with adjunctive rhu GM-CSF, and among five non-neutropenic patients with rhino-orbito-cerebral mucormycosis (three refractory to prior antifungal therapy) infection resolved in four, without recurrence over two to four years (Chen et al., 2022). Overall, these adjunctive approaches remain investigational. On current evidence, earlier diagnosis and consistent source control remain the most established means of improving outcome (Chamilos et al., 2008; Jeong et al., 2019a).

### 4.10 Outcomes and prognostic determinants

Our series is small, heterogeneous, and weighted toward critically ill patients. Descriptively, the six deaths occurred in patients with large surface-area burns, immunosuppression, advanced craniofacial disease or prolonged critical illness, which are features associated with higher mortality in larger cohorts (Rothe et al., 2021; Chen et al., 2023; Dang et al., 2023; Sigera and Denning, 2024; Cui et al., 2025; Skiada et al., 2025; Yang et al., 2025; Zia et al., 2025). The survivors, conversely, were immunocompetent trauma patients with localized, surgically accessible disease and one diabetic woman whose craniofacial disease, though severe, was ultimately brought under source control. This pattern is internally consistent with, but cannot independently confirm, the prognostic literature, in which reported associations with survival include host immune status, anatomical accessibility to surgery, and the timeliness of diagnosis and intervention (Sigera and Denning, 2024; Zia et al., 2025) and in which the best outcomes occur in localized cutaneous disease treated with combined modalities and the worst in disseminated or cerebral disease in the compromised host. Large recent cohorts continue to refine these determinants and to extend attention beyond mortality to functional and quality-of-life outcomes among survivors (Abdulkader et al., 2025; Zia et al., 2025).

## 5 Limitations

This study has several limitations. It is retrospective and based on a single center, and the number of patients is small. The nine cases were clinically diverse, differing in their underlying conditions and in the sites affected, so we cannot draw reliable conclusions about prognosis or about the relative value of the treatments used. Prognostic factors were thus not systematically assessed and the associations discussed in our Outcomes section are largely descriptive. Moreover, the high mortality should be read with the same caution, since most of our patients were critically ill and required intensive care. Iron indices and exposure to iron supplementation, transfusion or deferoxamine were not recorded, so the role of iron availability and siderophore-mediated iron acquisition could not be assessed. Molecular methods such as serum *Mucorales* PCR were not available to us during the study period. Their potential value is addressed in the Diagnosis subsection of the Discussion. Larger, multicentre studies would help define the true burden of disease in non-classical hosts, and could also assess early PCR screening in high-risk groups such as major burns, susceptibility-guided oral therapy, and adjunctive immunotherapy such as GM-CSF.

## 6 Conclusions

Mucormycosis can occur in patients without the classical predisposing factors such as uncontrolled DM, hematological malignancy, transplantation, or corticosteroid exposure. The predominance of traditional risk factors in the literature may be partly sustained by an epidemiological circularity, in which clinicians predominantly test for the disease where they already expect to find it. Breaking this diagnostic anchoring is crucial: a wound that becomes disproportionately necrotic, develops a thrombotic appearance, or fails to respond as expected to antibacterial treatment warrants tissue sampling for histopathology and fungal culture. In line with current international guidelines, L-AmB is the preferred agent, while isavuconazole is an option for cutaneous disease or as step-down therapy. Even so, in the profoundly immunosuppressed host, immediate and aggressive source control may not suffice, as the deficit that permits invasion is the one variable surgery cannot correct. The most important key learning points are summarized in **Figure 6**.

**Figure 6:**
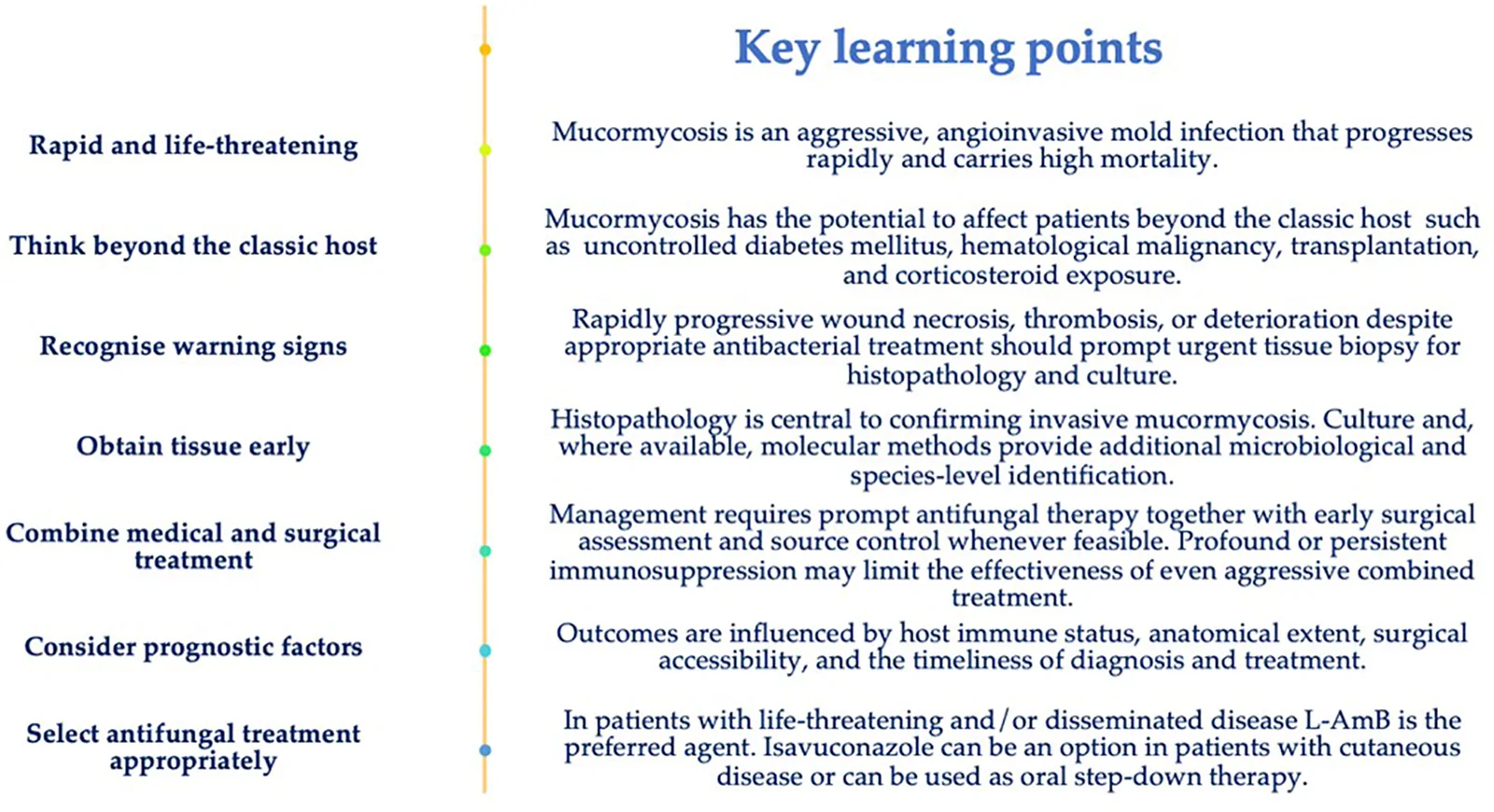
Key learning points.

## Data Availability

The original contributions presented in the study are included in the article/supplementary material. Further inquiries can be directed to the corresponding authors.
